# A new Python library to analyse skeleton images confirms malaria parasite remodelling of the red blood cell membrane skeleton

**DOI:** 10.7717/peerj.4312

**Published:** 2018-02-15

**Authors:** Juan Nunez-Iglesias, Adam J. Blanch, Oliver Looker, Matthew W. Dixon, Leann Tilley

**Affiliations:** 1 Melbourne Bioinformatics, The University of Melbourne, Melbourne, Australia; 2 Department of Biochemistry and Molecular Biology, Bio21 Institute, University of Melbourne, Melbourne, Australia

**Keywords:** Python, Skeleton analysis, Malaria, Red blood cell, Cytoskeleton

## Abstract

We present Skan (Skeleton analysis), a Python library for the analysis of the skeleton structures of objects. It was inspired by the “analyse skeletons” plugin for the Fiji image analysis software, but its extensive Application Programming Interface (API) allows users to examine and manipulate any intermediate data structures produced during the analysis. Further, its use of common Python data structures such as SciPy sparse matrices and pandas data frames opens the results to analysis within the extensive ecosystem of scientific libraries available in Python. We demonstrate the validity of Skan’s measurements by comparing its output to the established Analyze Skeletons Fiji plugin, and, with a new scanning electron microscopy (SEM)-based method, we confirm that the malaria parasite Plasmodium falciparum remodels the host red blood cell cytoskeleton, increasing the average distance between spectrin-actin junctions.

## Introduction

Skeletons are single-pixel thick representations of networks within an image, and have wide application to understanding the structural properties of objects. For example, skeletons have been used to model human poses, neuronal morphology, nanofibre structure, road networks, kidney development, and vascular networks, among others (Yim, Choyke & Summers, 2000; Sundar et al., 2003; Bas & Erdogmus, 2011; Yuan et al., 2009; Morales-Navarrete et al., 2015; Sambaer, Zatloukal & Kimmer, 2011). These applications include both 2D and 3D images, and often 3D images collected over time, underscoring the need for skeleton analysis software to support multiple imaging modalities and dimensionality.

In this paper, we report Skan, a Python library that produces graphs and branch statistics from skeleton images. Skan is written in Python using the Numba just-in-time (JIT) compiler (Lam, Pitrou & Seibert, 2015) for performance-critical code, including graph building and graph statistics computation. The source code is available at https://github.com/jni/skan (under a BSD 3-clause license), and we encourage readers to contribute code or raise GitHub issues where they require additional functionality to meet their needs. Skan can be installed using standard tools from the two leading Python repositories, the Python Package Index (PyPI) and conda-forge. Installation and usage instructions are available at https://jni.github.io/skan.

Skan works transparently with images of any dimensionality, allowing the analysis of 2D and 3D skeletons. Out of the box, Skan provides functions to compute the pixel skeleton graph, compute statistics about the branches of the skeleton, and draw skeletons and statistical overlays for 2D images.

The pixel skeleton graph maps which pixel is connected to which others in the skeleton image, as well as the distances between them. This graph is provided in the standard scipy.sparse.csr_matrix sparse matrix format, enabling further analysis using common tools for graph and array manipulation in the scientific Python ecosystem.

From this graph, we can compute statistics about the branches of the skeleton, defined as junction-junction and junction-endpoint paths in the pixel skeleton graph. These statistics include average branch length, branch type, branch curvature, branch endpoints, branch euclidean length, and average image intensity along the branch. We return these statistics as a pandas DataFrame, the de-facto standard format for data tables in Python. The table includes the pixel IDs of the branch endpoints, allowing further analysis of the junction-junction graph. Indeed, increasing the breadth of statistics computed by the software was the primary motivation for Skan’s development.

Skan further provides a rudimentary GUI to analyse batches of input images. The output of the GUI interface is an Excel file, which contains all the above-mentioned statistics, as well as all analysis parameters, to aid future reproducibility.

Because Skan uses common scientific Python data structures internally, it is easy to extend with new statistics and analyses. The DataFrame of branch statistics follows the “tidy data” paradigm (Wickham, 2014), with each row representing one branch of a skeleton, facilitating downstream analysis, such as computing summary statistics for each disjoint skeleton in an image.

To demonstrate Skan’s 3D capabilities, we first compared its output to that of Fiji’s Analyze Skeletons (Arganda-Carreras et al., 2010), applied to a publicly available dataset of neuron skeleton traces. Then, we used Skan to measure the spectrin cytoskeleton on the cytoplasmic side of the plasma membrane of red blood cells (RBCs) infected with the malaria parasite *Plasmodium falciparum*, using a new SEM-based protocol, and confirmed the remodelling of the RBC membrane skeleton by the parasite.

## Methods

### Analysis of skeleton model from DIADEM challenge

We downloaded the olfactory projection neuron 1 (OP-1) model as a SWC file from DIADEM’s website at http://diademchallenge.org/data_set_downloads.html, along with its corresponding 3D TIFF image stack. We then rasterised the model (i.e., converted it from a network of vertex coordinates to a set of active pixels) by using the Simple Neurite Tracer (Longair, Baker & Armstrong, 2011) plugin for Fiji, function “Analysis > Render/Analyze Skeletonized Paths.” This produces a 6-connected skeleton path, which we needed to convert to a (thinner) 26-connected path, so we further skeletonized the raster with the morphology.skeletonize3d function from scikit-image (Van der Walt et al., 2014), and saved it as a compressed TIFF file.

**Figure 1:**
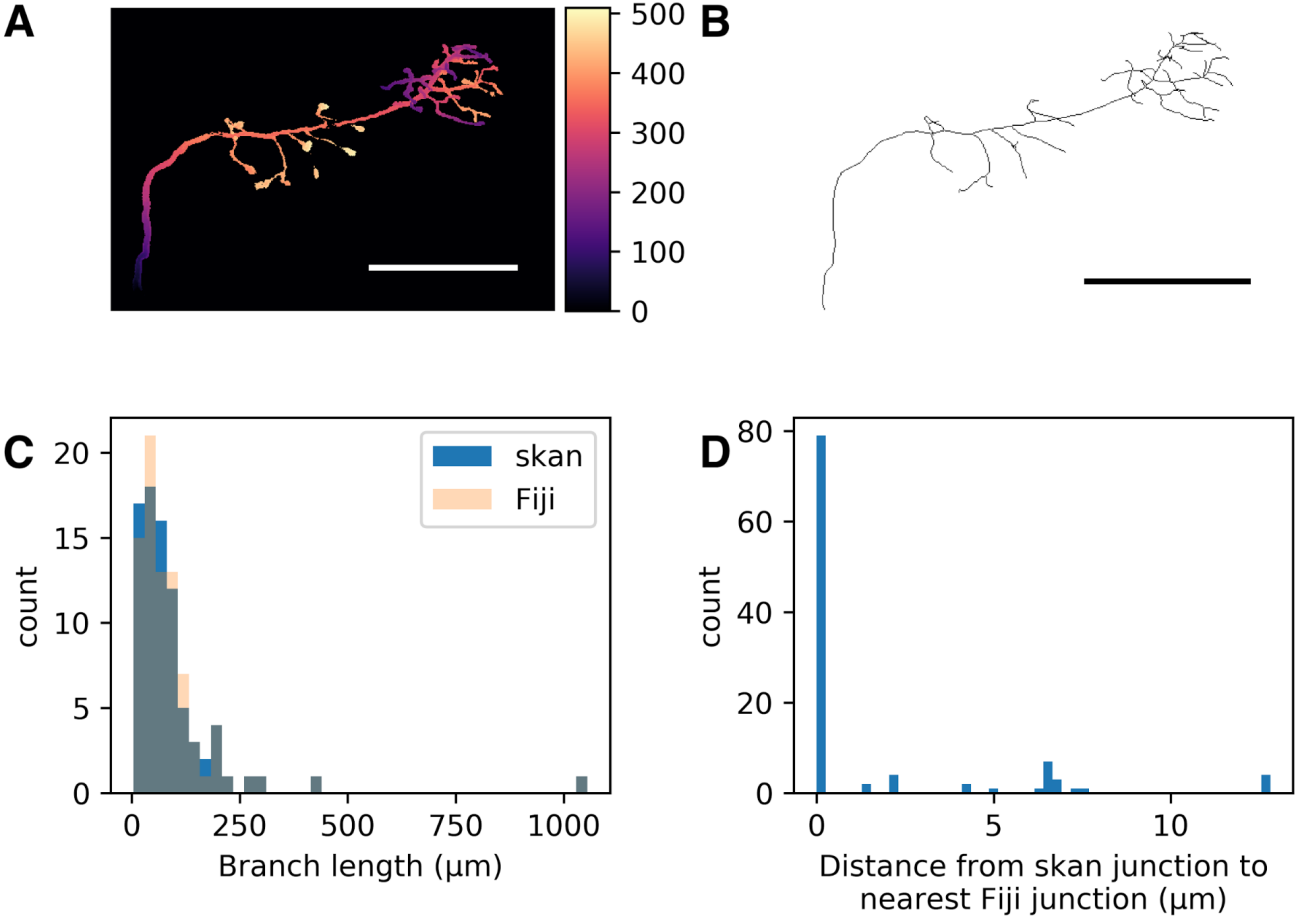
Comparison of skan and Fiji analysis results of the neuronal skeleton from olfactory projection neuron 1 (OP-1) from the DIADEM challenge. (A) Depth projection of the neuron. Scale bar: 500 μm. Colour map: height in μm. (B) Skeleton of the neuron. (C) Distribution of 82 branch lengths between 103 branch points measured by Skan and Fiji in the neuronal skeleton. (D) Distance from 103 skan junction points to the nearest Fiji junction point. Note that the voxel spacing is approximately 9 × 3 × 3 μm, so almost all of these distances are less than one pixel apart.

Then, we imported this raster image into either Fiji or Python (using Christoph Gohlke’s TIFF file). In both cases, we manually set the scale to 9.100602 × 3.033534 × 3.033534 μm per voxel, as documented on the DIADEM website. In Fiji, we used “Analyze Skeletons” with the “Show detailed info” option ticked, saved the results to csv, and loaded them into a pandas DataFrame in Python. In Python, we used skan.csr.summarise to produce a corresponding pandas DataFrame for Skan’s analysis. Finally, we used numpy.histogram and matplotlib.pyplot.hist (Hunter, 2007) to produce the histogram in Fig. 1.

### Tissue origin and ethics approval

This study made use of donated human red blood cells. All experiments were approved by The University of Melbourne School of Biomedical Sciences, Human Ethics Advisory Group (HEAG), for project titled “Characterising host cell interactions in the human malaria parasite, Plasmodium falciparum”, and ethics ID 1135799. Cells were obtained by a Material Supply Agreement with the Australian Red Cross Blood Service, agreement number—17-05VIC-23.

### Sample preparation and SEM imaging

To prepare sheared membranes, infected and uninfected red blood cells were attached to 3-Aminopropyl-triethoxysilane treated glass slides using the lectin erythro-agglutinating phytohemagglutinin (PHA-E) and sheared in a hypotonic buffer according to a previously established procedure (Shi et al., 2013). Sheared membranes were immediately fixed with 2.5% glutaraldehyde for 1 h before dehydration in a series of ethanol:water mixtures of 20, 50, 70, 80, 90, 95 and (3×) 100% ethanol for 5 min each and finally being allowed to dry in air.

Dried samples were gold coated on the rotating mount of a Dynavac SC100 sputter coating instrument for 35 s using a 25 mA current, measuring 0.2 nm thickness on the quartz crystal microbalance. The coating procedure was optimised to prevent under- or overcoating which presents problems with the skeleton trace.

SEM images were recorded using the ETD detector (in Optiplan mode) of an FEI Teneo instrument with a working distance of 5 mm, a beam current of 50 pA and a 2 kV accelerating voltage. Multiple images at 200–250 k magnification were recorded per individual cell to cover a greater portion of the membrane.

### Extraction of skeleton data from SEM images

In our SEM images, the spectrin-actin network appears as bright (raised) patches over dark patches of background (see Fig. 1). We followed a simple approach to trace the midline of the spectrin branches: smoothing the images, then thresholding them (Sauvola & Pietikäinen, 2000), and finally thinning them (Zhang & Suen, 1984). The width of the Gaussian smoothing, the window size for thresholding, and the offset for the thresholding are all parameters of our approach, and are recorded in the results output file of a skeleton analysis (when using the graphical user interface).

### Data and code availability

**Figure 2:**
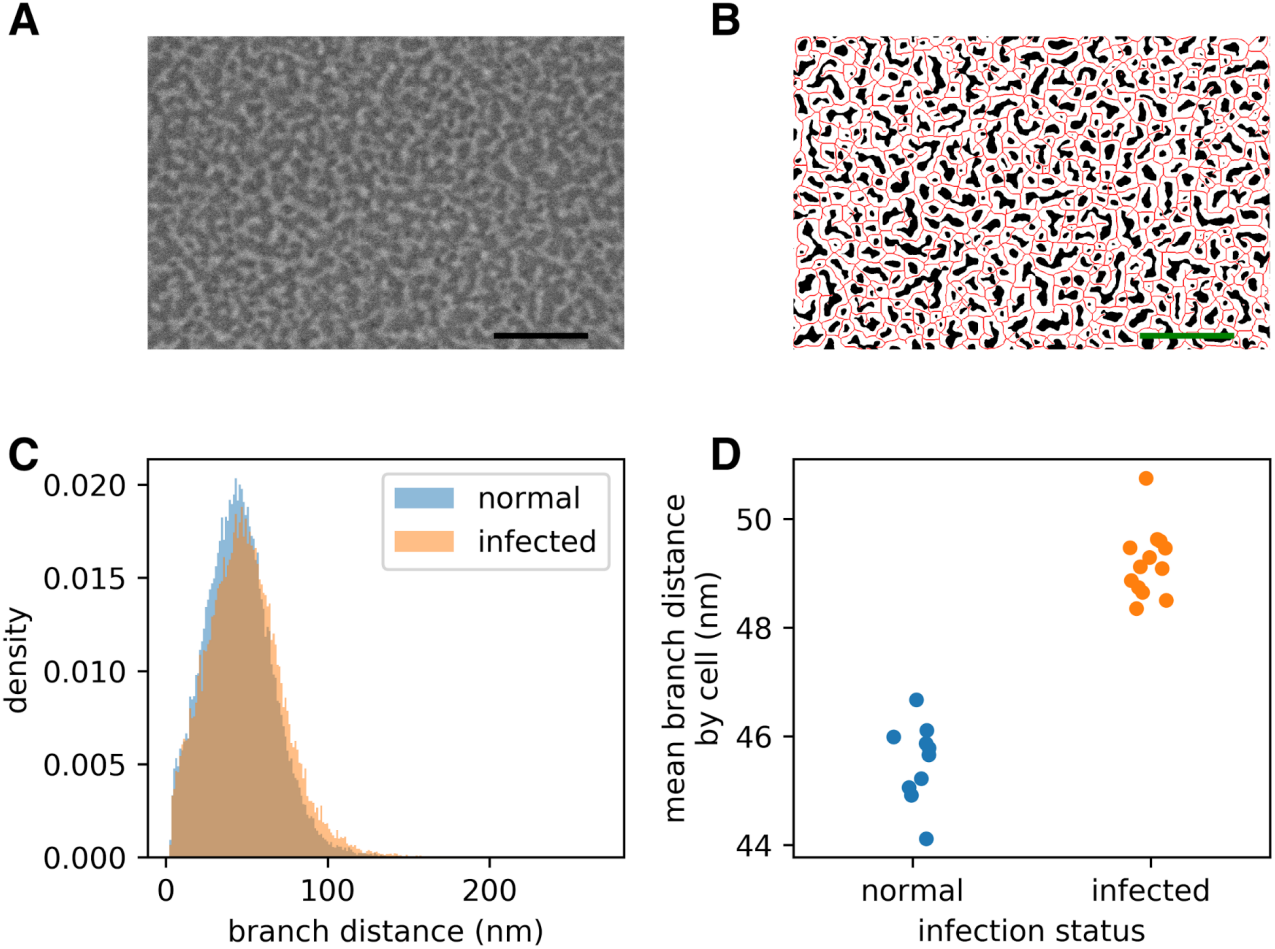
Infection by the malaria parasite remodels the spectrin skeleton of the host red blood cell in the asexual developmental stage. (A) Example image produced by our protocol. Scale bar: 300 nm. (B) Thresholding (white) and skeletonisation (red) of the image in (A). (C) Complete distribution of measured spectrin branch lengths for normal and infected RBCs. (D) Mean spectrin branch length by cell (*n*_norm_ = 10, *n*_inf_ = 13).

Our code is open source and available at https://github.com/jni/skan. Its documentation can be viewed at https://jni.github.io/skan and includes all code necessary to reproduce Fig. 2. Additional scripts used in our analyses are available at https://github.com/jni/skan-scripts.

We have made the schizont SEM dataset available at the Open Science Framework (OSF), with DOI 10.17605/OSF.IO/SVPFU, together with an archive of the documentation at time of publication, and a sample Excel file resulting from analysing the schizont dataset using the GUI.

## Results

### Comparison to Fiji’s analyze skeletons plugin

As a check that our software was producing results consistent with the existing literature on skeleton analysis, we compared our software’s results with that of Fiji’s Analyze Skeletons plugin (Arganda-Carreras et al., 2010). Although the original data from that paper is unavailable (I Arganda-Carreras, pers. comm., 2017), we compared the output of our software with that of Analyze Skeletons on a neuron skeleton from the DIADEM Challenge (http://diademchallenge.org) (Figs. 1A–1B). Both software packages found the same number of skeleton branches, with very close agreement between the two branch length distributions (Fig. 1C) and branch point locations (Fig. 1D). Manual inspection confirmed that the small differences observed result from the different treatment of branch junctions (see Supplementary information).

### Malaria parasites remodel the red blood cell inner membrane cytoskeleton

Prior studies have shown that infection by *P. falciparum*, the most deadly malaria-causing parasite, results in changes in the physical properties of the infected red blood cell (iRBC), and that these changes are associated with an elongation of the spectrin skeleton branches in the inner RBC membrane skeleton (Shi et al., 2013; Dearnley et al., 2016; Nans, Mohandas & Stokes, 2011). A coarse-grained molecular model suggested that this spectrin stretching could, in part, account for the deformability changes of the iRBC (Dearnley et al., 2016), emphasizing the biological significance of the measurements. We sought to confirm these observations using a novel scanning electron microscopy (SEM)-based protocol (A Blanch et al., 2017, unpublished data). The method involves cross-linking the RBCs to a glass coverslip and shearing off the upper membrane component, thus exposing the cytoplasmic/internal side of the cross-linked plasma membrane (Shi et al., 2013). The membrane is chemically fixed, dehydrated and gold-coated before imaging (Fig. 2A). We automatically extracted spectrin skeletons (Fig. 2B) from images produced using both uninfected RBCs and RBCs infected with mature stage parasites (40–44 h post infection). We found that the average spectrin branch distance increased from 45.5 nm to 49.2 nm, an increase of 8% (Figs. 2C–2D).

## Discussion and Conclusions

### Spectrin remodelling by *P. falciparum*

The remarkable deformability and durability of the RBC membrane derives from its membrane skeleton (Zhang et al., 2015). The skeleton is composed of a regular hexagonal array of “spring-like” proteins forming a meshwork at the cytoplasmic surface of the RBC. Spectrin heterodimers constitute the cross-beams of the molecular architecture and are connected to integral membrane proteins in the plasma membrane. Previous studies using atomic force microscopy (AFM) and transmission electron microscopy (TEM), followed by manual selection and measurement of skeleton branches, revealed reorganization and expansion of the spectrin network of the host cell membrane (Shi et al., 2013; Millholland et al., 2011; Cyrklaff et al., 2011).

In this work we have applied a novel SEM-based method to image the RBC membrane skeleton, and a fully automated method for selection and measurement of the spectrin branch distances. We observed an 8% increase in the length of the spectrin cross-members, in reasonable agreement with previous studies. Our data are consistent with the Cyrklaff et al. (2011) cryo-electron tomography study that provided evidence that the RBC membrane skeleton is reorganised as a result of mining of the actin junction points to generate actin filaments that connect parasite-derived organelles known as Maurer’s clefts to the knobs.

### Numba and performance

An interesting aspect of Skan’s development is its use of Numba, a just-in-time compiler for Python code. Skan is one of the first scientific packages to make extensive use of Numba to speed up its operations. In our hands, Numba has been able to dramatically speed up our code, in some cases approaching the theoretical maximum performance of our CPUs.

As just one example, in the context of implementing Sauvola image thresholding, we developed a function for the cross-correlation of an *n*-dimensional image with a sparse kernel. Sauvola thresholding requires computing the local mean and standard deviation for every pixel in an image. This can be optimally achieved by computing the integral of both the original image and the image of squared intensity values, and then convolving each with a kernel consisting of the outer product of the vector (−1, 0, 0, …, 0, 1) with itself, where the number of zeros is equal to the width of the neighbourhood minus one. This definition results in an extremely sparse kernel, which is not efficiently used by conventional convolution functions available in NumPy (v1.12) and SciPy (v0.19) (Van der Walt, Colbert & Varoquaux, 2011; Oliphant, 2007).

The function is implemented as correlate_sparse, which handles boundary conditions and formatting of the kernel, and then calls the Numba-jitted function _correlate_sparse_offsets, which iterates through the array, performing the cross-correlation.

The result is striking. For a 2,048 by 2,048 pixel image and a 31 by 31 kernel size, correlate_sparse takes 130 ms, somewhat slower than SciPy’s ndimage.correlate, which takes 57 ms. For a much bigger 301 by 301 kernel, however, correlate_sparse takes a similar amount of time—135 ms—while SciPy takes 17 s. Furthermore, if we analyse just the inner loop of the computation, the part handled by Numba, we measured a time of 1.8 ns per loop in our 1.3 GHz (i.e., 0.77 ns per cycle) CPU. Each loop performs two additions and a multiplication, in addition to array access, suggesting that Numba is close to achieving optimal performance for our problem and CPU. This example illustrates the power of Numba to speed up numerical Python code.

We also take this opportunity to note the loop order in the code of _correlate_sparse_offsets. For every non-zero element of the kernel, we make a full pass over the input image. When picturing a convolution, this is slightly counter-intuitive: most people would instead consider, for each pixel position, correlating all the non-zero elements of the kernel (thus examining each pixel only once).

However, that order of operations is poorly optimised for modern processor architectures, which fetch RAM contents by chunks into the processor cache. Once a chunk has been loaded, accessing elements of that chunk is 20–200 times faster than fetching more data from RAM (Jonas Bonér, https://gist.github.com/jboner/2841832). One consequence is that algorithms that access data in the order in which it is stored in RAM end up being much faster, by virtue of using processor cache to the maximum extent possible.

In our case, this translated to a 10-fold speedup when changing the order from (for pixel in image: for elem in kernel) to (for elem in kernel: for pixel in image), even though these two expressions are mathematically equivalent.

##  Supplemental Information

10.7717/peerj.4312/supp-1 Figure S1 Strategies for resolving skeleton junctions (A) A minimal skeleton. (B) Skan’s classification of pixels into endpoints, paths, and junctions based on the number of neighbours (1, 2, and 3 or more, respectively). (C) Identical classification in Fiji’s Analyze Skeletons. (D) Skeleton measurement when junctions are assigned an implicit “extent”. (E) Skeleton measurement when all adjacent junction pixels are replaced by their centroid (our default strategy). (F) Skeleton measurement used in Fiji’s Analyze skeletons (mid-2017 version).

10.7717/peerj.4312/supp-2 Supplemental Information 2 Supplementary results Pixel-level comparison with Analyze Skeletons.

## Additional Information and Declarations

### Data Availability

The following information was supplied regarding data availability: Code: https://github.com/jni/skan. Data: Open Science Framework public project, DOI 10.17605/OSF.IO/SVPFU.
https://osf.io/svpfu/.

## References

[ref-1] Arganda-Carreras I, Fernández-González R, Muñoz-Barrutia A, Ortiz-De-Solorzano C (2010). 3D reconstruction of histological sections: application to mammary gland tissue. Microscopy Research and Technique.

[ref-2] Bas E, Erdogmus D (2011). Principal curves as skeletons of tubular objects. Neuroinformatics.

[ref-3] Cyrklaff M, Sanchez CP, Kilian N, Bisseye C, Simpore J, Frischknecht F, Lanzer M (2011). Hemoglobins S and C interfere with actin remodeling in plasmodium falciparum—infected erythrocytes. Science.

[ref-4] Dearnley M, Chu T, Zhang Y, Looker O, Huang C, Klonis N, Yeoman J, Kenny S, Arora M, Osborne JM, Chandramohanadas R, Zhang S, Dixon MWA, Tilley L (2016). Reversible host cell remodeling underpins deformability changes in malaria parasite sexual blood stages. Proceedings of the National Academy of Sciences of the United States of America.

[ref-5] Hunter JD (2007). Matplotlib: a 2D graphics environment. Computing in Science & Engineering.

[ref-6] Lam SK, Pitrou A, Seibert S (2015). Numba: a LLVM-based python JIT compiler.

[ref-7] Longair MH, Baker DA, Armstrong JD (2011). Simple Neurite Tracer: open source software for reconstruction, visualization and analysis of neuronal processes. Bioinformatics.

[ref-8] Millholland MG, Chandramohanadas R, Pizzarro A, Wehr A, Shi H, Darling C, Lim CT, Greenbaum DC (2011). The malaria parasite progressively dismantles the host erythrocyte cytoskeleton for efficient egress. Molecular & Cellular Proteomics.

[ref-9] Morales-Navarrete H, Segovia-Miranda F, Klukowski P, Meyer K, Nonaka H, Marsico G, Chernykh M, Kalaidzidis A, Zerial M, Kalaidzidis Y (2015). A versatile pipeline for the multi-scale digital reconstruction and quantitative analysis of 3D tissue architecture. eLife.

[ref-10] Nans A, Mohandas N, Stokes DL (2011). Native ultrastructure of the red cell cytoskeleton by cryo-electron tomography. Biophysical Journal.

[ref-11] Oliphant TE (2007). SciPy: Open source scientific tools for Python. Computing in Science and Engineering.

[ref-12] Sambaer W, Zatloukal M, Kimmer D (2011). 3D modeling of filtration process via polyurethane nanofiber based nonwoven filters prepared by electrospinning process. Chemical Engineering Science.

[ref-13] Sauvola J, Pietikäinen M (2000). Adaptive document image binarization. Pattern Recognition.

[ref-14] Shi H, Liu Z, Li A, Yin J, Chong AGL, Tan K. SW, Zhang Y, Lim CT (2013). Life cycle-dependent cytoskeletal modifications in Plasmodium falciparum infected erythrocytes. PLOS ONE.

[ref-15] Sundar H, Silver D, Gagvani N, Dickinson S (2003). Skeleton based shape matching and retrieval.

[ref-16] Van der Walt S, Colbert SC, Varoquaux G (2011). The NumPy array: a structure for efficient numerical computation. Computing in Science & Engineering.

[ref-17] Van der Walt S, Schönberger JL, Nunez-Iglesias J, Boulogne F, Warner JD, Yager N, Gouillart E, Yu T, Scikit-image contributors (2014). Scikit-image: image processing in Python. PeerJ.

[ref-18] Wickham H (2014). Tidy data. Journal of Statistical Software.

[ref-19] Yim PJ, Choyke PL, Summers RM (2000). Gray-scale skeletonization of small vessels in magnetic resonance angiography. IEEE Transactions on Medical Imaging.

[ref-20] Yuan X, Trachtenberg JT, Potter SM, Roysam B (2009). MDL constrained 3-D grayscale skeletonization algorithm for automated extraction of dendrites and spines from fluorescence confocal images. Neuroinformatics.

[ref-21] Zhang Y, Huang C, Kim S, Golkaram M, Dixon MWA, Tilley L, Li J, Zhang S, Suresh S (2015). Multiple stiffening effects of nanoscale knobs on human red blood cells infected with Plasmodium falciparummalaria parasite. Proceedings of the National Academy of Sciences of the United States of America.

[ref-22] Zhang TY, Suen CY (1984). A fast parallel algorithm for thinning digital patterns. Communications of the ACM.

