# Supplementary figures and images for: A new Python library to analyse skeleton images confirms malaria parasite remodelling of the red blood cell membrane skeleton
Source: PeerJ. 2018 Feb 15;6:e4312. doi: 10.7717/peerj.4312 (PMC5816961; doi:10.7717/peerj.4312)

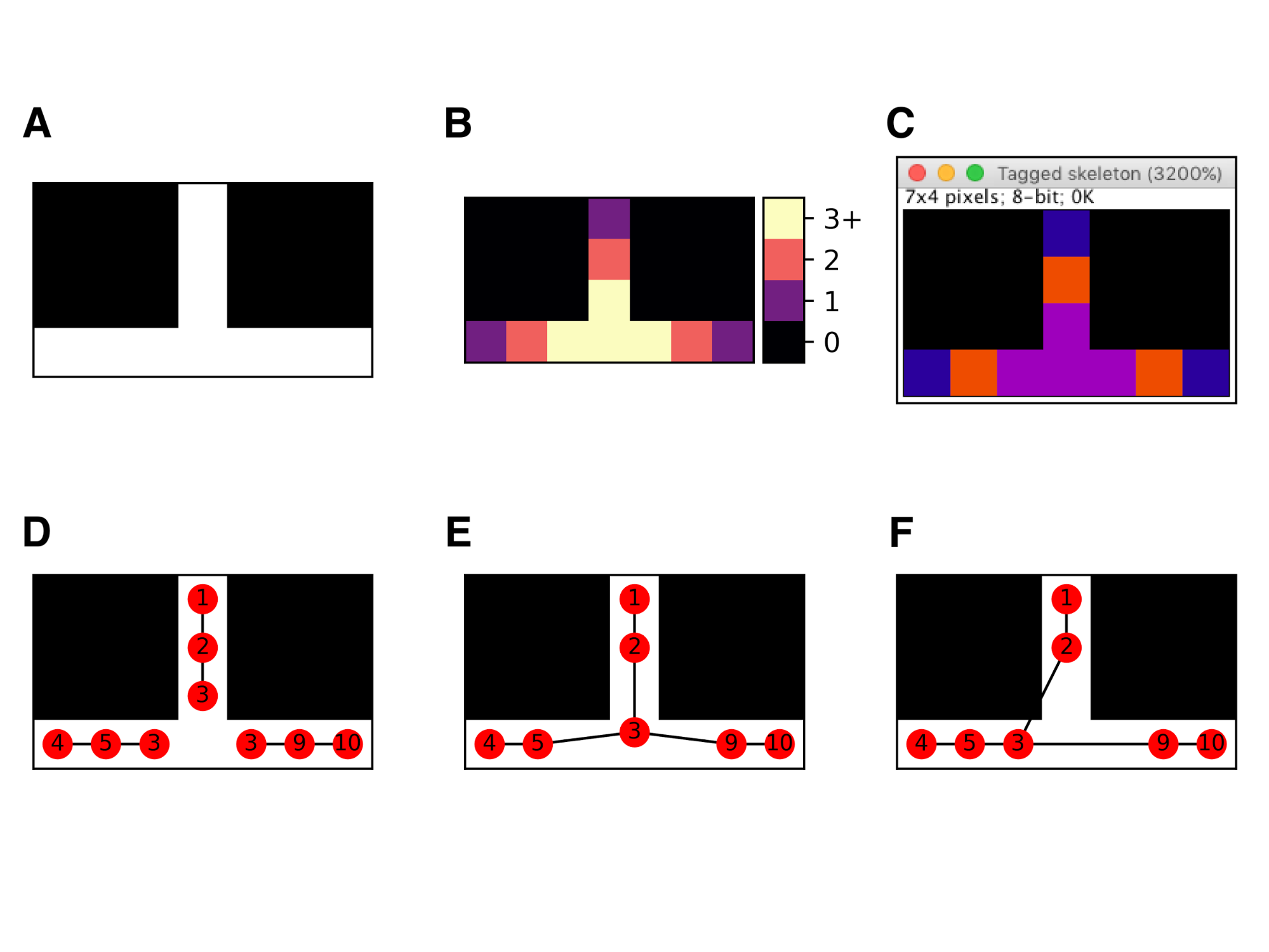

Supplement: Figure S1 — (A) A minimal skeleton. (B) Skan’s classification of pixels into endpoints, paths, and junctions based on the number of neighbours (1, 2, and 3 or more, respectively). (C) Identical classification in Fiji’s Analyze Skeletons. (D) Skeleton measurement when junctions are assigned an implicit “extent”. (E) Skeleton measurement when all adjacent junction pixels are replaced by their centroid (our default strategy). (F) Skeleton measurement used in Fiji’s Analyze skeletons (mid-2017 version). [file peerj-06-4312-s001.png]
