# Supplementary material for: A new Python library to analyse skeleton images confirms malaria parasite remodelling of the red blood cell membrane skeleton
Source: PeerJ. 2018 Feb 15;6:e4312. doi: 10.7717/peerj.4312 (PMC5816961; doi:10.7717/peerj.4312)
Supplement: Supplemental Information 2 — Pixel-level comparison with Analyze Skeletons. [file peerj-06-4312-s002.pdf]

SUPPLEMENTARY RESULTS

Pixel-level comparison with Analyze Skeletons

Although the results obtained by our software are broadly similar to those of Analyze Skeletons, we investigated the behaviour of both tools on a tiny example image, to ensure that we understood the minor discrepancies between the two.

The differences between the libraries occur in the handling of clusters of junction nodes. It is impossible to guarantee that several branches of a skeleton will converge on a single “junction” pixel. For example, Supplementary Figure 1A shows a fully-reduced skeleton of 10 pixels. Counting the number of neighbours of each pixel, we classify pixels as end-points, paths, and junctions. However, we can see that four contiguous pixels at the bottom-centre are all considered junctions (Supplementary Figure S1B-C). We saw three possible ways to deal with such clusters when computing branch statistics:

1. Ignore them — the branch from pixel 1 to pixel 3 will be considered to have length 2, as will the other branches; the junction clusters are thus considered to have some “spatial extent.” (Supplementary Figure S1D)

2. Replace them by a single pixel, perhaps the pixel closest to the centroid of the pixels.

3. Replace them by their centroid. (Supplementary Figure S1E)

In Skan, we added a flag, `unique_junctions`, that selects between options 1 and 3. The Analyze Skeletons plugin, in contrast, uses a variant of option 2, where the pixel selected to represent the cluster is the “earliest,” in lexicographical order of (x, y[, z]) position (Supplementary Figure S1F). Although in most situations these small subtleties would make little difference to the downstream results, we believe our approach is closer to what a user would expect to get from their skeleton images.

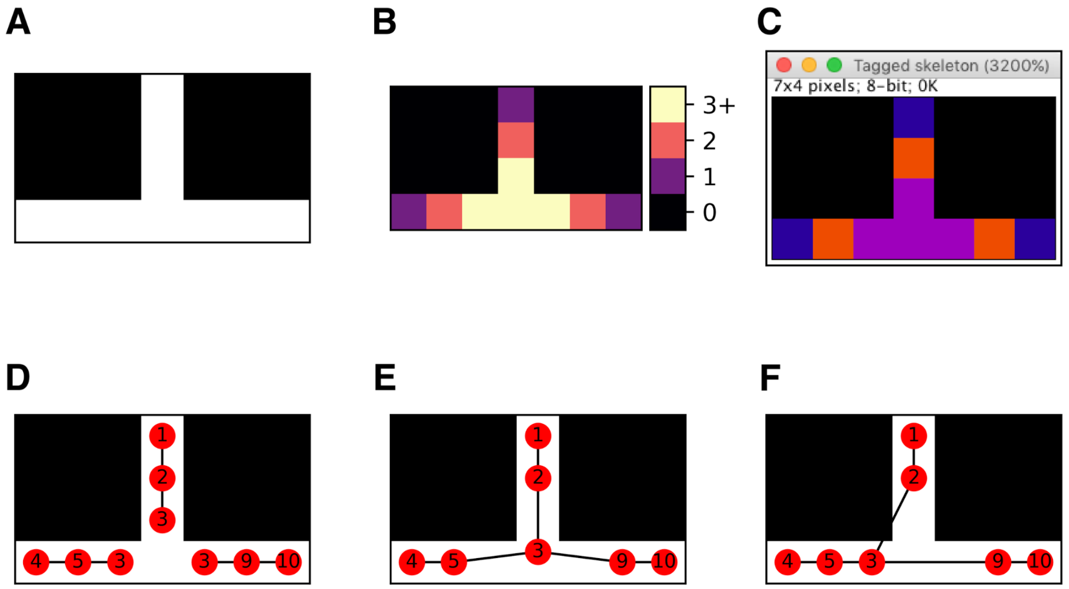

Figure S1. Strategies for resolving skeleton junctions. (A) A minimal skeleton. (B) Skan's classification of pixels into endpoints, paths, and junctions based on the number of neighbours (1, 2, and 3 or more, respectively). (C) Identical classification in Fiji's Analyze Skeletons. (D) Skeleton measurement when junctions are assigned an implicit "extent". (E) Skeleton measurement when all adjacent junction pixels are replaced by their centroid (our default strategy). (F) Skeleton measurement used in Fiji's Analyze skeletons (mid-2017 version).
